# Type 1 diabetes alters early macrophage-*Mycobacterium tuberculosis* transcriptional coordination during infection

**DOI:** 10.1016/j.isci.2026.116418

**Published:** 2026-06-23

**Authors:** Julia Brake, Nicholas A. Sumpter, Valerie A.C.M. Koeken, Jodie A. Schildkraut, Eva Terschlüsen, Cees J. Tack, Edwin Ardiansyah, Vinod Kumar, Jakko van Ingen, Reinout van Crevel

**Affiliations:** 1 Department of Internal Medicine and Radboudumc Community for Infectious Diseases, Radboud University Medical Center, Nijmegen, the Netherlands; 2 Research Centre Innovations in Care, Rotterdam University of Applied Sciences, Rotterdam, the Netherlands; 3 Department of Pulmonary Diseases, Pharmacology and Toxicology, Radboud University Medical Center, Nijmegen, the Netherlands; 4 Department of Medical Microbiology, Radboud University Medical Center, Nijmegen, the Netherlands; 5 Research Center for Care and Control of Infectious Diseases, Universitas Padjadjaran, Bandung, Indonesia; 6 Nitte University Centre for Science Education & Research, Paneer Campus, Deralakatte, Mangalore, India; 7 Blizard Institute, Faculty of Medicine & Dentistry, Queen Mary University of London, London, UK

**Keywords:** health sciences, medicine, immunology

## Abstract

Diabetes mellitus is a risk factor for tuberculosis, but the underlying mechanisms remain unclear. We examined the host-*Mycobacterium tuberculosis* (*Mtb*) interaction in diabetes. Monocyte-derived macrophages from people with type 1 diabetes and healthy controls were infected with *Mtb*, and analyzed for host and *Mtb* gene expression and culture supernatant cytokines. Expression of antibacterial defense genes upon infection was mostly similar, but IFN-γ, both at RNA and protein level, was lower in macrophages from people with diabetes. Intracellular *Mtb* showed stress responses and a metabolic shutdown, both in macrophages from people with diabetes and controls. Expression of *CYBB* and other Mendelian susceptibility to mycobacterial disease genes strongly correlated with expression of *Mtb* cell wall and lipid genes in control macrophages, but much less so in diabetes. This rich dual RNA-seq dataset shows how type 1 diabetes may affect cross-talk between macrophages and *Mtb*, providing a valuable resource for future mechanistic studies.

## Highlights

• Dual RNA-seq shows type 1 diabetes macrophage response to *Mtb* largely mirrors healthy controls
• Intracellular *Mtb* shows stress response and metabolic shutdown in all macrophages
• IFN-γ upregulation is slightly dampened on gene and protein level in type 1 diabetes
• Macrophage *CYBB* and mycobacterial cell wall and lipid gene correlation is weaker in diabetes

## Graphical abstract

**Figure:**
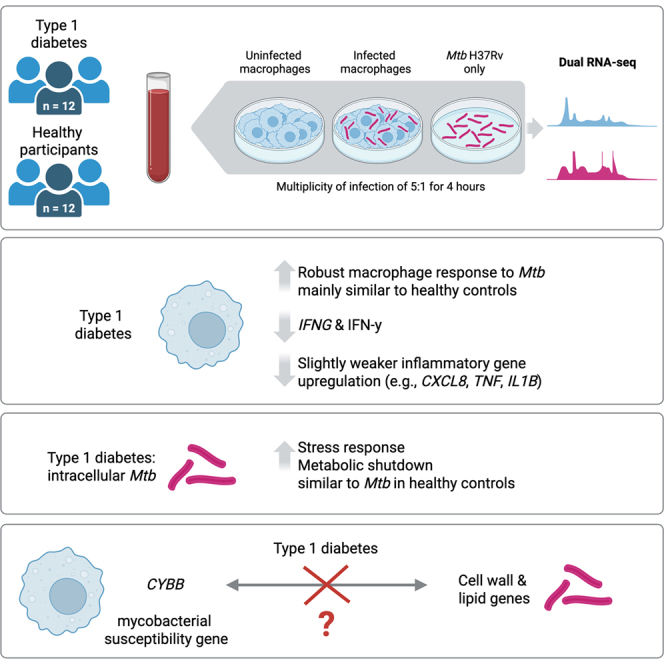

## Introduction

Diabetes mellitus (DM) significantly increases the risk of developing active tuberculosis (TB),^1^ with 15% of TB cases attributed to diabetes globally.^2^ While both type 1 and type 2 diabetes associate with increased TB risk,^3^^,^^4^^,^^5^^,^^6^ type 1 diabetes may confer more risk when poorly controlled.^7^ Among TB cases, diabetes is associated with increased bacterial burden,^6^^,^^8^ more pulmonary cavities,^9^ and more treatment failure and recurrences.^10^^,^^11^ Epidemiological evidence strongly links diabetes to TB, yet the mechanisms underlying this effect remain unclear.

Macrophages provide the main niche for *Mycobacterium tuberculosis* (*Mtb*) and serve as an effector cell that eliminates *Mtb*.^10^ Macrophage origin influences the environment for *Mtb* and therefore likely determines infection outcomes.^11^^,^^12^^,^^13^ The microenvironment in people with diabetes exposes macrophages to hyperglycemic conditions, and may alter the host response to *Mtb*, with evidence for decreased phagocytosis, altered cytokine production, and higher reactive oxygen species (ROS) as well as lower nitric oxide (NO) production.^14^^,^^15^ These data suggest that macrophages from people with diabetes interact differently with *Mtb*.

Dual RNA-sequencing (dual RNA-seq) enables simultaneous profiling of gene expression of both host and pathogen, providing insights into their interactions during infection.^11^^,^^16^^,^^17^^,^^18^^,^^19^^,^^20^ However, generating dual RNA-seq data poses significant challenges due to the low amount of bacterial relative to host RNA. This often results in constrained mycobacterial read counts and a high sequencing depth of host RNA. While a few studies have successfully applied dual RNA-seq to investigate macrophage-pathogen interactions,^11^^,^^16^^,^^17^^,^^18^^,^^19^^,^^20^ revealing for instance ontologically distinct macrophage-*Mtb* interactions,^11^ most dual RNA-seq studies focused scarcely on *Mtb* and exclusively on healthy macrophages.

In this study, we performed dual RNA-seq to investigate the transcriptional interaction of macrophages from people with diabetes and healthy controls with *Mtb*. We hypothesized that differences in the interaction between host and *Mtb* in diabetes may contribute to increased susceptibility.

## Results

### *Mtb* infection induces strong transcriptional response in macrophages from people with diabetes and controls

**Figure 1:**
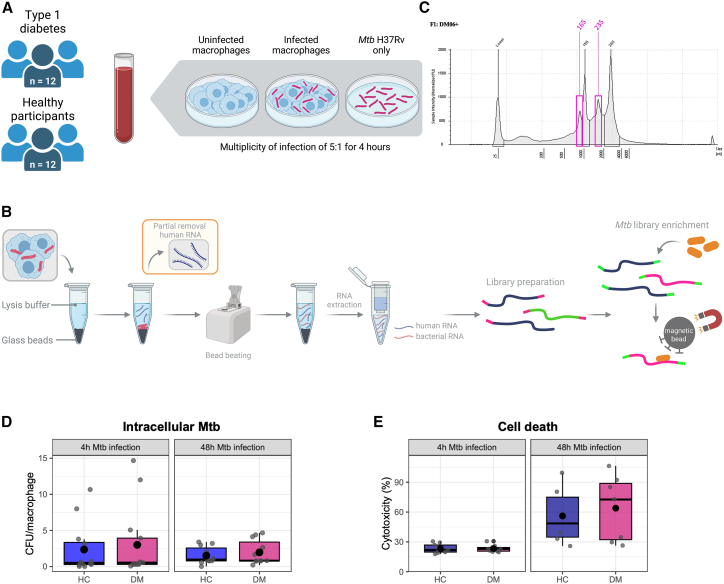
Dual RNA-sequencing workflow for the enrichment of mycobacterial RNA from human macrophages (A) Schematic representation of the study design. Blood was collected from each participant, followed by monocyte isolation and differentiation into human-monocyte derived macrophages for dual RNA-sequencing of uninfected macrophages, *Mtb*-infected macrophages, and *Mtb* H37Rv alone. (B) For RNA isolation, macrophages were first incubated with a lysis buffer, with release of human RNA. To enrich for bacterial RNA and libraries, 60% of human RNA volume was removed, followed by bead-beating to release bacterial RNA. Customized MyBaits probes were used to specifically enrich *Mtb* libraries for further amplification. (C) Representative figure of ribosomal RNA subunit peaks (gel electrophoresis) to measure RNA integrity on an Agilent Bioanalyzer 2100. Extracted RNA contained visible ribosomal RNA subunit peaks of both the prokaryotic 16 S and 23 S rRNA molecules (pink) and eukaryotic 18 S and 28 S rRNA molecules (gray). (D) Intracellular *Mtb* counts (colony forming units/macrophage) after four and 48 h post *Mtb* infection in macrophages from people with diabetes (pink, 4 h, *n* = 13; 48 h, *n* = 11) and healthy controls (blue, 4 h, *n* = 12; 48 h, *n* = 11). (E) Cytotoxicity (%) measured by lactate dehydrogenase release after four and 48 h post *Mtb* infection in macrophages from people with diabetes (pink, 4 h, *n* = 9; 48 h, *n* = 7) and healthy controls (blue, 4 h, *n* = 9; 48 h, *n* = 6) compared to a 100% lysis control. (D and E) No significant differences between phenotypes were detected (median = black horizontal boxplot line, mean = black bubble, two-sided Wilcoxon rank-sum test; unadjusted *p* value < 0.05).

**Figure 2:**
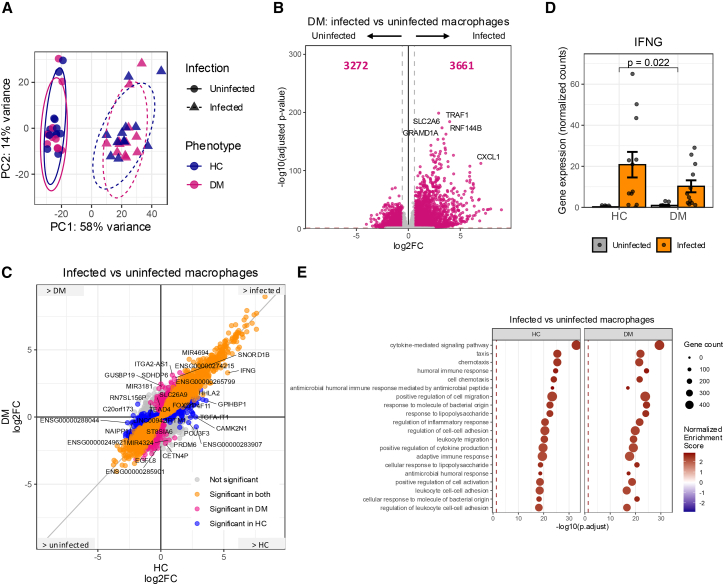
Transcriptional response of macrophages from people with diabetes and controls to *Mtb* infection (A) Principal-component analysis (PCA) plot showing the first two principal components of measured gene expression derived from infected (triangles) and uninfected (circles) macrophages of people with diabetes (pink, *n* = 11) and healthy control subjects (blue, *n* = 12). (B) Volcano plot comparing gene expression of infected and uninfected macrophages from people with diabetes (*n* = 11), showing significance (–log10 P) versus magnitude of change (Log2 fold change) of differentially expressed genes. Significant differences between infection state are depicted in pink (DeSeq2 design formula: ∼Sample ID + Infection; adjusted *p* value < 0.05, log2FC > 0.585). (C) Combined Volcano plot displaying the Log_2_ fold change between infected and uninfected macrophages for both macrophages from people with diabetes (*n* = 11) and controls (*n* = 12). Significant changes between infected and uninfected macrophages are shown for diabetic macrophages (pink), healthy macrophages (blue), and both groups (orange), with labeling of genes with the largest log2FC difference between macrophages from people with diabetes and controls (DeSeq2 design formula: ∼Sample ID + Infection; adjusted *p* value < 0.05). (D) Bar chart showing normalized gene counts for IFNG in uninfected (gray) and infected (orange) macrophages from people with diabetes (DM) and healthy controls (HCs) (DeSeq2 design formula: ∼Phenotype + Phenotype:ID_special + Phenotype:Infection; unadjusted *p* value < 0.05, not corrected for multiple testing). (E) GSEA using permutation testing showing the 20 most enriched pathways upon infection for both DM and HC, ranked by significance (–log10[adjusted *p* value]). The red dotted line represents the threshold of significance (adjusted *p* value < 0.05). Size of the bubbles reflects the gene ratio of the pathway (hit gene count/total gene count) and the color reflects the normalized enrichment score (red = positive, blue = negative).

Macrophages from people with type 1 diabetes (*n* = 12) and healthy controls (*n* = 12, Table 1) were infected with *Mtb*, followed by dual RNA-seq of host and pathogen RNA (Figures 1A–1C). *Mtb* infection resulted in similar intracellular *Mtb* counts and macrophage cell death from people with diabetes and healthy controls (Figures 1D and 1E). We first examined how *Mtb* infection affects the macrophage transcriptome and whether the presence of diabetes was associated with different macrophage transcriptional responses. Macrophage transcriptomes clearly differed based on the infection status, which explained 58% of variance between samples in the first principal component (PC) (Figure 2A). PC2 separated samples of individual experiments and PC3 clearly separated samples based on sex (Figure S2C). Macrophages from people with diabetes did not clearly differ from controls in the principal components. *Mtb* infection resulted in substantial transcriptional changes in both macrophages from people with diabetes and controls (Figures 2B and S2D). These changes were largely similar between both macrophage groups. Two-thirds (64%) of DEGs overlapped in significance between macrophages from people with diabetes and controls upon *Mtb* infection (Figure S2H), 16% of genes were only differentially expressed in control macrophages (Figure S2H). The latter belonged to pathways of cellular signaling and response to stimulus (Figure S2I). 20% of genes were only differentially expressed in macrophages from people with diabetes (Figure S2H). However, only few of these non-overlapping genes were statistically different between macrophages from people with diabetes and controls (Figures S2E and S2F), with a minor read count difference between infection states (Figure S2G). Also, when examining the effect of diabetes on infection outcomes using an interaction model, we found no significant differences (data not shown).

Of the genes that were differentially expressed in both macrophage groups upon infection, there was some evidence for differences in effect size and gene ranks between macrophages from people with diabetes and controls. In macrophages from people with diabetes *TRAF1*, *SLC2A6* and a pseudogene were the three most upregulated genes (Figures S3A and S3B), whereas *CXCL8* and *TNF*, the most upregulated genes in control macrophages, ranked as number 62 and 48 respectively in macrophages from people with diabetes. Some genes showed different fold change expression between the two macrophage groups, including *ITGA2-AS1*, *SNORD1B*, *GUSBP19*, *SLC26A9*, *TEAD4*, *EGFL8*, *NAIPP1*, and *IFNG* (Figure 2C). The largest log2FC difference of 3.5 was found for an understudied transcript called *ENSG00000283907*. Interestingly, *IFNG* was 32 times higher upon infection compared to baseline in control macrophages, while only six times higher in macrophages from people with diabetes, showing a five times greater upregulation in control macrophages. *IFNG* upregulation upon infection significantly differed between macrophages from people with diabetes and controls when tested independently. This was due to both a slight increase in uninfected macrophages from people with diabetes, and an approximately two times lower final count after infection compared to control macrophages (Figure 2D).

Upon infection, both macrophages from people with diabetes and controls showed enrichment of antimicrobial pathways, such as cytokine-mediated signaling, chemotaxis, humoral and adaptive immune response, inflammatory response, and cell/leukocyte activation pathways (Figure 2E). Antimicrobial response and chemotaxis pathways were slightly more enriched in control macrophages, while macrophages from people with diabetes showed stronger negative enrichment in sensory perception and chemical stimulus and slightly greater enrichment in apoptotic signaling (Figure S3C).

### Secreted cytokines reflect the *Mtb-*induced transcriptional response of macrophages

**Figure 3:**
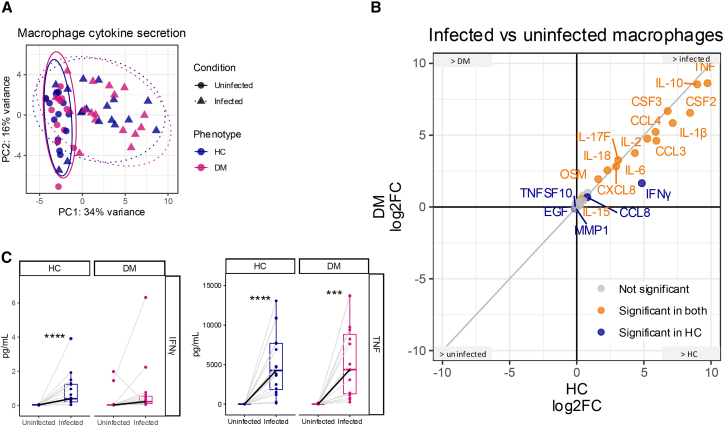
Cytokine secretion of macrophages from people with diabetes and controls upon *Mtb* infection (A) PCA plot showing the first two principal components of measured cytokines derived from infected (triangles) and uninfected (circles) macrophages from people with diabetes (pink, uninfected, *n* = 15; infected, *n* = 17) and healthy controls (blue, uninfected, *n* = 16; infected, *n* = 17). (B) Combined Volcano plot displaying the Log_2_ fold change between paired infected and uninfected macrophages from people with diabetes (*n* = 15) and controls (*n* = 16). Significant changes between infected and uninfected control macrophages (blue) and both macrophage groups (orange) are displayed (two-sided paired Wilcoxon rank-sum test; adjusted *p* value < 0.05). (C) Boxplots showing the absolute concentrations (pg/mL) of IFN-y and TNF in supernatants of paired infected and uninfected macrophages from people with diabetes (pink, *n* = 15) and controls (blue, *n* = 16) (two-sided paired Wilcoxon rank-sum test; adjusted *p* value < 0.05). ∗ adj. *p* < 0.05, ∗∗ adj. *p* < 0.01, ∗∗∗ adj. *p* < 0.001.

We next measured cytokines in macrophage supernatants comparing individuals with (*n* = 17) and without diabetes (*n* = 17). Supernatant cytokine profiles correlated with infection status, explaining 34% of the variance between samples (Figure 3A). Most proteins, including TNF, IL-10, CSF2/3, IL-1β, CCL3/4, IL-2, IL-6, IL-17 F, CXCL8, IL-18, and IL-15 were significantly upregulated in macrophages of both groups upon *Mtb* infection (Figures 3B, S4B, and S4C). Interestingly, control macrophages showed a 5-fold stronger increase of IFN-γ production upon infection with *Mtb* compared to macrophages from people with diabetes (Figures 3B and 3C). Taken together, cytokine responses to *Mtb* infection were generally similar in macrophages from people with diabetes and controls, except for IFN-γ secretion, which is in line with the transcriptional difference.

### *Mtb* transcriptionally adapts to the intracellular macrophage environment

**Figure 4:**
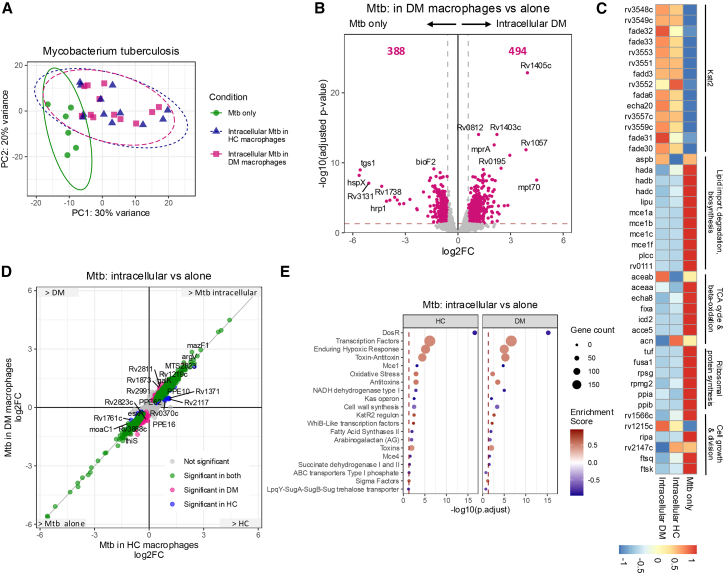
Transcriptional response of intracellular *Mtb* in macrophages from people with diabetes and controls (A) PCA plot showing the first two principal components of measured gene expression derived from *Mtb* alone (green, *n* = 6), intracellular *Mtb* in macrophages from people with diabetes (pink, *n* = 12) or healthy controls (blue, *n* = 12). (B) Volcano plot comparing gene expression of *Mtb* alone (*n* = 6) versus being intracellular in macrophages from people with diabetes (*n* = 12), showing significance (–log10 P) versus magnitude of change (Log2 fold change) of differentially expressed genes, with significant differences depicted in pink (DeSeq2 design formula: ∼Condition; adjusted *p* value < 0.05, log2FC > 0.585). (C) Heatmap representing the *z*-scores of the mean of the three groups for genes belonging to specific bacterial processes, such as cholesterol catabolism (Kstr2), lipid metabolism, and other metabolic processes (*z* scores > 0 = red, <0 = blue). (D) Combined Volcano plot displaying the Log_2_ fold change in gene expression of intracellular *Mtb* compared to *Mtb* alone (*n* = 6). Significant differences between intracellular *Mtb* and *Mtb* alone in macrophages from people with diabetes (pink, *n* = 12), controls (blue, *n* = 12), or both groups are displayed (green; DeSeq2 design formula, ∼Condition; adjusted *p* value < 0.05). (E) GSEA showing the 20 most enriched pathways upon *Mtb* being intracellular in DM or HC, ranked by significance (–log10[adjusted *p* value]). The red dotted line represents the threshold of significance (adjusted *p* value < 0.05). Size of the bubbles reflects the gene ratio of the pathway (hit gene count/total gene count) and the color reflects the normalized enrichment score (red = positive, blue = negative). Pathways derive from the CAMM categories.

We next examined the transcriptional response of intracellular *Mtb* in macrophages from people with and without diabetes. The difference between *Mtb* transcriptomes was mainly explained by the infection status (Figure 4A). *Mtb* significantly upregulated 420 genes and downregulated 278 genes upon being intracellular in healthy control macrophages (Figure S5A). Inside macrophages from people with diabetes, *Mtb* significantly upregulated 494 genes and downregulated 388 genes (Figure 4B). Most *Mtb* genes were similarly expressed in macrophages from people with diabetes and controls, with no significant DEGs identified between both groups (Figures 4D and S5B). Also ranks of upregulated *Mtb* genes mostly overlapped between macrophages from people with diabetes and controls, such as for *Rv1405c* and *Rv1403c*, methyltransferases relevant for adaptation to intracellular stressors,^21^ *Rv0812*, *mprA*, *Rv1057*, *IppA*, *mpt70*, *furA*, and *vapC37* (Figures S5E and S5F). Two-thirds (69%) of DEGs reached significance inside both macrophage groups, 6% of *Mtb* genes were only differentially expressed inside control macrophages, and 25% only in macrophages from people with diabetes (Figure S5D).

In both macrophages from people with diabetes and controls, *Mtb* gene expression of the Kstr2 regulon, important for cholesterol degradation to support bacterial survival, was strongly increased upon infection (Figure 4C). Different from that, genes involved in lipid metabolism, the TCA cycle, beta-oxidation, ribosomal protein synthesis, and cell growth were downregulated in both intracellular *Mtb* groups (Figure 4C). Gene set enrichment analysis (GSEA) showed that the most positively enriched pathways belonged to transcription factors, enduring hypoxic response, toxin-antitoxin, oxidative stress, Kstr2 regulon, and WhiB-Like transcription factors (Figure 4E). The most negatively enriched pathways were DosR, Mce1, NADH dehydrogenase type I, cell wall synthesis, kas operon, fatty acid synthases II, arabinogalactan, Mce4, ABC transporters type I phosphate, and succinate dehydrogenase I and III (Figure 4E).

There were minor differences between intracellular *Mtb* gene expression in macrophages from people with diabetes and controls. The log2FC of all intracellular *Mtb* genes was overall slightly higher inside macrophages from people with diabetes compared to controls (Figure S5C). Genes with the biggest difference in log2FC between being in macrophages from people with diabetes or healthy controls included *PPE10*, *PPE16*, *thiS*, *moaC1*, *esxP*, and *galK* (Figure 4D). However, overall, intracellular *Mtb* exhibited a similar response upon entering the macrophage environment, whether in macrophages from people with diabetes or healthy controls.

### Host MSMD genes show genome-wide transcriptional coordination with intracellular *Mtb*

As a final step we correlated host and *Mtb* transcriptomes. As described above, macrophage transcriptional responses were largely similar between people with diabetes and healthy control individuals, with *IFNG* emerging as the most consistent difference between groups. Given that genetic defects in *IFNG*-linked pathways underlie “Mendelian susceptibility to mycobacterial disease” (MSMD), we first examined how MSMD-associated host genes correlated with intracellular *Mtb* transcriptional patterns.

**Figure 5:**
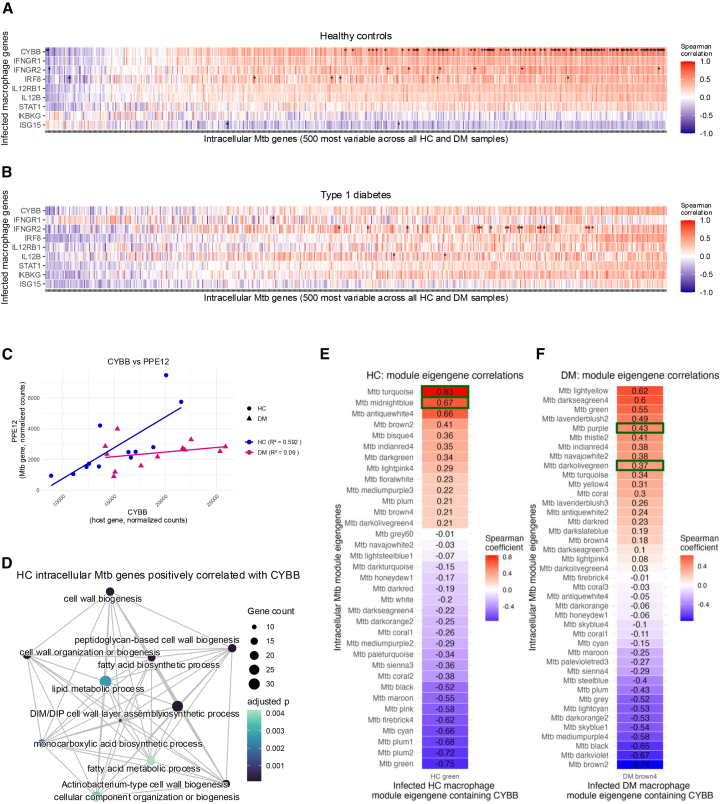
Transcriptional interaction of macrophages with *Mtb* during infection (A and B) Heatmaps displaying the FDR-corrected Spearman’s rank correlation coefficient with of normalized gene count expression of MSMD-associated genes from infected (A) healthy control macrophages or (B) macrophages from people with diabetes versus the 500 most variable genes across all intracellular *Mtb* samples (correlation coefficient >0 = red, <0 = blue; adjusted *p* value < 0.05). (C) Linear regression for gene expression of the intracellular *Mtb* gene (*PPE12*), which most strongly correlated with the host gene expression of *CYBB* in infected control macrophages. Regression lines are shown for *CYBB* of infected macrophages from people with diabetes (pink, triangle) and controls (blue, circle) versus their respective intracellular *Mtb* expression of *PPE12*. Coefficient of determination (R^2^) is shown in the legend. (D) Gene overrepresentation analysis of intracellular *Mtb* genes, which correlated with *CYBB* expression in infected healthy control macrophages (spearman correlation coefficient >0.6), ranked by significance (–log10[adjusted *p* value]). The red dotted line represents the threshold of significance (adjusted *p* value < 0.05). Size of the bubbles reflects the gene ratio of the pathway (hit gene count/total gene count), and the color reflects the percentage of genes present in the pathway, ranging from 0% (dark blue) to 100% (yellow). Pathways derive from the GO database. (E and F) Spearman’s correlation of the module eigengene (ME) from infected (E) healthy (*n* = 12) or (F) macrophages from people with diabetes (*n* = 12) versus module eigengenes from the respective intracellular *Mtb* genes. Module eigengenes derived from a WGCNA. Co-expression modules were calculated for all host genes and separately for all intracellular *Mtb* genes per phenotype. The displayed host module eigengenes (HC green and DM brown4) derive from the module that contained the gene *CYBB*. All module eigengenes from the WGCNA analysis of intracellular *Mtb* genes are displayed on the *x* axis. Intracellular *Mtb* module eigengenes, which contain most lipid and cell wall related genes that correlated positively with *CYBB* in Figure 5A are highlighted with a green box. (Correlation coefficient >0 = red, <0 = blue; adjusted *p* value < 0.05).

First we correlated the expression of nine MSMD genes^22^ with 500 *Mtb* genes with the most variable intracellular gene expression, which likely represent those that are under strongest pressure from the macrophage environment. Using Spearman rank correlation adjusted for multiple testing, several MSMD-related host genes, including *CYBB*, *IFNGR1*, *IFNGR2*, *IRF8*, *IL12RB1*, *IL12B*, and *STAT1*, showed predominantly positive correlations with *Mtb* gene expression in control macrophages (Figures 5A and S6A). Notably, *CYBB* was significantly positively correlated with 98 *Mtb* genes, while *IFNGR2* and *IRF8* correlated significantly with six and five *Mtb* genes, respectively (Figure 5A). In contrast, *ISG15* showed a small number of significant negative associations (Figure 5A).

MSMD genes also correlated with variable intracellular *Mtb* gene expression in macrophages from people with diabetes, but different from controls (Figure 5B). Specifically, positive correlations between *CYBB* and *Mtb* genes were attenuated, whereas *IFNGR2*, *IL12B*, *IKBKG*, and *ISG15* demonstrated relatively stronger or more frequent positive correlations with *Mtb* genes compared to control macrophages (Figures 5A, 5B, and S6A). At the level of individual host-pathogen gene pairs, one of the strongest correlations observed in control macrophages was between *CYBB* and multiple *Mtb* PPE family genes; these correlations were markedly weaker in macrophages from people with diabetes (Figure 5C). The *CYBB*-correlated *Mtb* genes belonged to cell wall biogenesis and lipid metabolism pathways (Figure 5D), including among others some genes of the PPE family (*PPE12*, *PPE13*, *PPE18*, *PPE19*, *PPE2*, *PPE21*, *PPE22*, *PPE24*, *PPE3*, *PPE34*, *PPE35*, *PPE36*, *PPE4*, *PPE5*, *PPE53*, *PPE54*, *PPE55*, *PPE56*, *PPE57*, *PPE58*, *PPE59*, *PPE6*, *PPE62*, *PPE64*, and *PPE8*), fatty acyl AMP-ligases (*fadD2*, *fadD21*, *fadD22*, *fadD23*, *fadD25*, *fadD26*, *fadD28*, *fadD29*, *fadD30*, *fadD31*, *fadD32*, *fadD5*, and *fadE8*), polyketide synthases (*pks4*, *pks6*, *pks7*, *pks8*, *pks9*, and *pks12*), ESX/type VII secretion system genes (*eccA2*, *eccA5*, *eccC5*, *eccCa1*, *eccCb1*, *eccE5*, *efpA*, *espB*, *espC*, *espE*, *espF*, *espI*, *espR*, *esxK*, *esxL*, *esxN*, and *esxP*), cell envelope lipoproteins (*lppJ*, *lppU*, *lppX*, *lpqM*, *lipN*, and *ltp1*), and lipid and mycolic acid transporters (*mmpL2*, *mmpL3*, *mmpL4*, *mmpL5*, *mmpL8*, *mmpL9*, *mmpL10*, and *mmpL12*).

We next evaluated host-pathogen coordination in an unbiased, genome-wide manner. We applied weighted gene co-expression network analysis (WGCNA) independently to the host and intracellular *Mtb* transcriptomes and defined modules of co-expressed genes (Figures S6D and S6E). Module eigengenes were then correlated to assess coordination between host and pathogen transcriptional programs at a network level. This showed that only few host and *Mtb* modules showed significant correlation (Figures S6F and S6G). Interestingly, in control macrophages the module “green” containing *CYBB* showed the strongest correlation with the *Mtb* modules “turquoise” and “midnightblue” (Figure 5E), which included 84 and 15 of the *Mtb* genes that positively correlated with *CYBB*, respectively, (coefficient >0.6) in Figure 5A. *CYBB* was well-aligned with its module’s expression pattern (kME of 0.81). In macrophages from people with diabetes, the strong correlation between the *CYBB*-containing host module “brown4” (*CYBB* kME of 0.56) and the *Mtb* modules with most cell wall and lipid metabolism genes (“darkolivegreen” [33 genes] and “purple” [26 genes]), was lost (Figure 5F), highlighting the unique coordination in healthy control macrophages.

Together, these analyses demonstrate that MSMD/*IFNG*-axis host responses are among the strongly coordinated with intracellular *Mtb* transcription (both at gene and co-expressed gene module level), but that this host-pathogen transcriptional coupling is somewhat diminished in macrophages from people with diabetes.

## Discussion

We performed dual RNA-seq of *Mtb-*infected macrophages from individuals with and without diabetes at an early infection time point. *Mtb* infection induced strong transcriptional responses in all macrophages, but IFN-γ upregulation was decreased in cells from people with diabetes, both at RNA and protein level. The transcriptional response of intracellular *Mtb* was largely similar in macrophages from people with diabetes and controls and characterized by upregulation of stress responses and metabolic shutdown. When integrating macrophage and *Mtb* gene expression, diabetes was associated with a diminished correlation of host MSMD immune genes and *Mtb* genes related to cell wall and lipid metabolism.

Macrophages from people with diabetes and controls mostly responded similarly to early *Mtb* infection with upregulation of antimicrobial pathways. This may be explained by the fact that type 1 diabetes was mostly well controlled, because *in vitro* differentiation of primary monocytes may have led to a convergence of macrophage phenotypes, because of M-CSF use, standard medium glucose concentration of 11 mM and use of human pooled rather than autologous serum, although cross-serum experiments have shown no effect of serum from people with diabetes on cytokine profiles of peripheral blood mononuclear cells (PBMCs).^23^ Other studies have observed that macrophages from people with diabetes retain an immunological imprint even when placed in a different environment^24^^,^^25^ and that monocyte-derived macrophages from individuals with diabetes (and TB) differ in cytokine profiles,^26^ activation markers,^27^ and gene expression of M1/M2 cytokines/markers,^28^ indicating that host phenotype differences persist during macrophage differentiation. In line with our findings, other studies have found no similar *Mtb*-induced immune cell responses in healthy individuals and those with type 1 diabetes. For example, cellular metabolism and pattern recognition expression in PBMCs exposed to *Mtb* showed no difference.^23^ Similarly, macrophage differentiation under hyperglycaemia did not show differences in *Mtb* killing, outgrowth, or phagocytosis, though cytokine responses were altered at extreme glucose concentrations.^29^ Of course, negative findings on this topic are much less likely to have been published. Still, other studies have reported an altered response of macrophages from people with diabetes to *Mtb*, including dysregulated pattern recognition receptors, decreased phagocytosis, and altered cytokine production.^14^^,^^28^^,^^30^^,^^31^^,^^32^^,^^33^ The divergence between studies might represent variation in diabetes characteristics or *in vitro* methods.

We identified some interesting differences in macrophage responses upon *Mtb* infection. Notably, the IFN-γ response was reduced in macrophages from people with diabetes, both at a transcriptional and protein level. Reduced IFN-γ has been previously detected in macrophages from people with diabetes and diabetes-TB co-morbidity compared to people with TB alone.^28^ IFN-γ is crucial for the response against *Mtb*.^34^^,^^35^ It is primarily produced by lymphocytes and natural killer cells, stimulating macrophages to enhance microbicidal activities, including phagosome maturation, production of ROS, NO, and antigen presentation.^36^ Residual lymphocytes could theoretically have contributed to IFN-γ levels, but macrophage cultures were thoroughly washed, making this less likely. Mouse alveolar macrophages have shown production of IFN-γ upon *Mtb* and IL-12 stimulation,^37^^,^^38^ but human macrophages also secrete IFN-γ.^39^^,^^40^^,^^41^^,^^42^^,^^43^ Macrophage-secreted IFN-γ is also biologically active.^40^ In our study, the secreted IFN-y protein levels are relatively low compared to T cell-secreted IFN-γ, but others have detected similarly low IFN-γ levels at this early time point.^43^ Moreover, several studies show that longer duration of stimulation leads to increased IFN-γ secretion by macrophages,^38^^,^^40^^,^^43^ indicating that maximal IFN-γ levels and potentially biologically relevant concentrations might not have been reached yet. Next to controlling intracellular *Mtb* growth by inducing microbicidal activities, IFN-γ enhances cell death of heavily infected macrophages.^44^ Macrophage endogenously secreted IFN-γ might thus act as an additional macrophage activation signal and/or cell death promoter, and lower IFN-γ levels in diabetes may lead to more *Mtb* intracellular growth. Also, macrophages from people with diabetes showed a slightly lower magnitude of expression for host response genes in the nuclear factor-kappa B (NF-κB) signaling pathway, including *TNF*, *CXCL8*, and *IL1B*.^45^^,^^46^ This suggests that the early macrophage response may be subtly impaired, leading to reduced macrophage activation and lower levels of *IFNG* in type 1 diabetes. This hypothesis should be mechanistically validated in future studies.

*Mtb* gene expression was strongly affected upon early intracellular infection of both macrophages from people with type 1 diabetes and controls. Intracellular *Mtb* showed transcriptional stress responses and a metabolic shutdown. Once phagocytosed by macrophages, *Mtb* is exposed to several host defenses, such as hypoxia, nutritional immunity, ROS, NO, and an acidic pH in phagosomes.^47^ We observed upregulation of the enduring hypoxic response *Mtb* pathway, which contributes to induction of dormancy.^48^ The dormancy survival regulator (DosR) induces genes relevant for persistence, such as *tgs1* to accumulate lipid bodies as energy source.^49^^,^^50^ DosR was downregulated in our study, possibly explained by the early 4 h infection time point. Some DosR genes have been shown to be downregulated after 4 h of starvation^51^ and only upregulated after 24 h under hypoxic conditions and NO when stimulated with IFN-γ.^52^ Moreover, the mammalian cell entry (Mce) 1 operon proteins (e.g., *mce1A*-*mce1F*, *yrbE1A*, and *YrbE1B*), present in the cell wall, are involved in cell entry, mycolic acid recycling, lipid transport, and pro-inflammatory macrophage responses.^53^^,^^54^ We assume that *Mce1* levels decrease after a first peak relevant for cell entry as shown after 24 h of starvation.^51^ Cell wall membrane pathways and the Kas operon, which is important for mycolic acid synthesis,^55^ were most likely downregulated to reduce energy-consuming processes. Reduced NADH dehydrogenase 1 expression reflects the switch from aerobic to anaerobic respiration as it is involved in the oxygen-dependent electron transport chain.^56^ Additionally, downregulation of genes involved in metabolic pathways, such as the TCA cycle, reflect the overall growth arrest to adjust to the host environment.^57^ Notably, in line with other studies,^11^ one of the most upregulated pathways was the transcriptional regulator (Ketoacyl-ACP synthase 2) Kstr2, which controls genes involved in cholesterol uptake and degradation.^58^ Host lipid utilization is essential for *Mtb* to survive during infection^59^^,^^60^^,^^61^^,^^62^ and this displays the utilization of macrophage cholesterol to support survival.

Finally, when looking at correlation of host and *Mtb* gene transcription during early infection, expression of *CYBB* and other MSMD genes, crucial for an effective host immune response against *Mtb*,^22^ strongly correlated with expression of certain *Mtb* virulence genes. This coordination between the host and *Mtb* was less clear in macrophages from people with diabetes, which suggests that diabetes either lowers the protective macrophage response to these *Mtb* virulence factors, or that *Mtb* shows less of a virulence response to key host defense mechanisms in macrophages from people with diabetes. Notably, macrophages from people with diabetes showed slightly higher baseline *CYBB* levels but less upregulation upon *Mtb* infection compared to control macrophages, possibly reflecting increased systemic inflammation in individuals with type 1 diabetes^63^ and a lower ability to respond upon infection, as observed for *IFNG*.

Interestingly, *CYBB* expression in healthy control macrophages correlated with intracellular *Mtb* expression of sets of *PPE* genes as well as *fadD* and *pks* genes. *PPE* proteins have three distinct functions in *Mtb*. First, with their high number, variety, and cell wall localization,^64^ they are thought to increase antigenic variation and thereby immune evasion.^65^ Second, *PPE* genes are involved in immunomodulatory mechanisms; for example, *PPE18*, *PPE19*, and *PPE60* have been shown to increase adhesion to and uptake by macrophages,^66^ while *PPE2* secretion dampens ROS and NO production by macrophages,^67^ and *PPE13* activates the inflammasome.^68^ Third, *PPE* proteins, including *PPE51*, are involved in nutrient uptake.^69^ However, the role of most *PPE* genes has yet to be determined. *FadD* genes encode fatty acyl-CoA ligases and fatty acyl-AMP ligases active in lipid biosynthesis as well as catabolism; for lipid biosynthesis they activate fatty acids and transfer them to polyketide synthases (*pks*).^70^^,^^71^ *FadD* genes synthesize virulence lipids for the *Mtb* cell envelope. For example, *FadD29* activates fatty acids for phthiocerol dimycocerosate synthesis,^72^ which structurally protects against microbicidal factors and which is essential for immune evasion by for instance phagosomal escape.^73^^,^^74^ Together, this suggests that *CYBB* expression in macrophages is associated with expression of adaptational response systems in *Mtb*, including both modulation of the microbe-macrophage interaction as well as metabolic reprogramming with a shift to lipid metabolism. These appear dampened in macrophages from people with type 1 diabetes during early infection.

Our results are hypothesis-generating and the described interaction of *Mtb* with host response genes like *CYBB* could be unraveled further by use of CRISPR-Cas mutated *Mtb* strains, either reducing or increasing expression of *Mtb* virulence or metabolic genes, and comparing responses in macrophages from people with diabetes or controls. Overall, increased TB susceptibility in well controlled type 1 diabetes is unlikely to arise from a fundamentally impaired macrophage response to *Mtb* during early infection. Instead, mild dysregulation of IFN-γ signaling and subtle disruption of host-pathogen gene co-expression need further investigation.

### Limitations of the study

Our study has several limitations. First, the *Mtb* strain H37Rv was used. Being a laboratory-adapted strain, H37Rv has lost some virulence factors and lacks genetic diversity which may affect macrophage responses. Nevertheless, use of the same strain across experiments ensures comparability between macrophages from people with diabetes and healthy controls, minimizing variability that could determine observed differences. Second, macrophages are the main niche for *Mtb*, but other immune cells are involved as well, and whole blood or single-cell analysis may better reveal how diabetes affects macrophage function within the broader immune context. Third, the 4 h infection period was possibly too short to detect metabolic differences between macrophages from people with diabetes and controls in light of the strong early effect of *Mtb* infection. However, early host-pathogen interaction is critical for the outcome of infection. Finally, besides measuring cytokine secretion, we did not functionally validate associations between host and *Mtb* transcriptome. Our dual RNA-seq study of *Mtb* infections however provides a large data resource, and the only one using macrophages derived from people with diabetes. To further refine the model, future studies might also include *Mtb* isolates from individuals with diabetes which have shown genomic differences,^75^ or metabolic *Mtb* mutants.

## Resource availability

### Lead contact

Requests for further information and resources should be directed to and will be fulfilled by the lead contact, Julia Brake (julia.brake@radboudumc.nl).

### Materials availability

There were no new materials generated in this study.

### Data and code availability

• All data and code are deposited and publicly available at GitHub:
• https://github.com/juliabrake/Dual_RNAseq (https://doi.org/10.5281/zenodo.20345705)
  ◦ Read count tables of dual RNA-sequencing results (CountTableHuman.xlsx and CountTableMtb.xlsx).
  ◦ Metadata of human participants and *Mtb* samples (MetadataHuman.xlsx and MetadataMtb.xlsx).
  ◦ Cytokine data (DuSe_Olink_Data_Meta.xlsx).
  ◦ Code developed for the dual RNA-seq data analysis (DuSe_Analysis_JB.qmd).
  ◦ Code developed for cytokine data and additional analyses (DuSe_Olink_Julia.qmd).
  ◦ Code developed for WGCNA analysis (DuSe_Analysis_JB WGCNA.qmd).
• Any additional information required to reanalyze the data reported in this study is available upon request from the lead contact.

## Acknowledgments

We would like to thank all participants. Moreover, we thank Liesbeth van Emst for performing the Olink measurement in-house, Hanna Fricke for helping with the bacterial pathway analysis, Rinke Stienstra for discussing data, Elizabeth A. Wynn and Nicholas D. Walter for providing the CAMM categories, Michiel Oorsprong for navigating the sequencing process, and Helena van der Steege for her help with the cytotoxicity assay experiments. J.B. and R.v.C. are supported by the 10.13039/501100009708Novo Nordisk Foundation (grant number NNF25SA0104395) and the EU (RIA2018CO-2514-PROTID), and R.v.C. by the EU and Romanian government (PNRR/2023/C9/MCID/I8. contract no. 760273/26.03.2024). The graphical abstract was created using Biorender (Brake, J., 2026; LZ29MQ6RH8).

## Author contributions

Conceptualization and design of the study, J.B., V.A.C.M.K., J.A.S., and R.v.C.; participant inclusion, C.J.T. and J.B.; sample collection and experimental performance, J.B. and E.T.; data analysis, J.B., N.A.S., E.A., and V.K.; data interpretation, J.B., N.A.S., J.v.I., and R.v.C.; writing – original draft, J.B.; review and editing, R.v.C. and J.B.; reading and approval of final version, all authors. All authors had full access to the data and were responsible for deciding to submit it for publication.

## Declaration of interests

The authors declare no competing interests.

## STAR★Methods

### Key resources table

**Table:** 

REAGENT or RESOURCE	SOURCE	IDENTIFIER
**Bacterial and virus strains**		

*Mycobacterium tuberculosis* H37Rv strain	ATCC	Cat#27294

**Biological samples**		

Human blood samples	Study participants	

**Critical commercial assays**		

Olink Target 48 Cytokine panel	Olink®	https://olink.com/products/olink-target-48
NucleoSpin RNA isolation Kit	Macherey-Nagel	Cat#740955.50
Illumina Stranded Total RNA Prep, Ligation with Ribo-Zero Plus	Illumina	Cat#20040529
IDT for Illumina RNA UD Indexes Set A, Ligation	Illumina	Cat#20040553
MyBaits Kit Custom 100-120 K (48 rxn/kit)	Arbor Biosciences	Not applicable; customized
Qubit RNA High Sensitivity (HS)	Thermo Fisher	Cat#Q32852
Qubit dsDNA BR Assay Kit	Thermo Fisher	Cat#Q33230
High Sensitivity RNA ScreenTape and sample buffer	Agilent	Cat#5067-5579 Cat#5067-5580
D1000 ScreenTape and sample buffer	Agilent	Cat#5067-5582 Cat#5067-5602
Glucose assay kit	Abcam	Cat#ab65333
Protein/CRP DuoSet ELISA	Bio-Techne R&D Systems	Cat#DY1707

**Deposited data**		

Read count tables of dual RNA-sequencing results	This study	https://github.com/juliabrake/Dual_RNAseq (CountTableHuman.xlsx and CountTableMtb.xlsx) https://doi.org/10.5281/zenodo.20345705
Metadata of human participants and *Mtb* samples	This study	https://github.com/juliabrake/Dual_RNAseq (MetadataHuman.xlsx and MetadataMtb.xlsx) https://doi.org/10.5281/zenodo.20345705
Cytokine data	This study	https://github.com/juliabrake/Dual_RNAseq (DuSe_Olink_Data_Meta.xlsx) https://doi.org/10.5281/zenodo.20345705

**Oligonucleotides**		

Index primer Illumina i7: CAAGCAGAAGACGGCATACGA	Sigma-Aldrich	Cat#8823472352-10/0
Index primer Illumina i5: AATGATACGGCGACCACCGA	Sigma-Aldrich	Cat#8823472352-20/0

**Software and algorithms**		

Code developed for the dual RNA-seq data analysis	This paper	https://github.com/juliabrake/Dual_RNAseq (DuSe_Analysis_JB.qmd) https://doi.org/10.5281/zenodo.20345705
Code developed for cytokine data and additional analyses	This paper	https://github.com/juliabrake/Dual_RNAseq (DuSe_Olink_Julia.qmd) https://doi.org/10.5281/zenodo.20345705
Code developed for weighted gene co-expression network analysis	This paper	https://github.com/juliabrake/Dual_RNAseq (DuSe_Analysis_JB WGCNA.qmd) https://doi.org/10.5281/zenodo.20345705
R V.4.5.0	The R Foundation for Statistical Computing	R: The R Project for Statistical Computing

### Experimental model and study participant details

#### Participant inclusion

Individuals with type 1 diabetes (*n* = 17) and healthy controls (*n* = 17), matched by age and sex, were included. Exclusion criteria were systemic medication use, such as immunosuppressive drugs like corticosteroids (except oral contraceptives), pregnancy, breastfeeding, kidney replacement therapy or renal insufficiency (eGFR <50), recent illness or vaccination (within the previous 2 weeks), immunodeficiency, or other co-morbidities that caused significant inflammation or metabolic disturbance, such as cancer or serious infections. Data collected included age, sex, height, weight, and smoking status. Type 1 diabetes participants had a diagnosis for at least 7 years. Additionally, data about diabetes duration, HbA1c, insulin and other medication use, and diabetes-related complications were collected.

#### Study participants

Study subjects were not tested for tuberculosis, because this is extremely rare in the Netherlands (incidence 3/100.000/year),^76^ with *Mtb* infection prevalence <1% among those born after 1960. Routine BCG vaccination was discontinued in the Netherlands in 1979 and is now limited to children with one or both parents born in a country with a TB incidence > 50/100.000. None of the volunteers belonged to that group. Twelve individuals from each group were selected for dual RNA-seq based on the quality of RNA (Figures 1A; Table 1). All individuals with type 1 diabetes and healthy controls were sex- and age- matched with an average age of 46 and 34 years respectively and five out of 17 female participants. Individuals with type 1 diabetes had a higher BMI (26.1 kg/m^2^ vs. 23.5 kg/m^2^) and higher systemic inflammatory C-Reactive Protein levels (3.62 mg/L vs. 1.44 mg/L) (Table S2). Individuals with type 1 diabetes had a mean HbA_1c_ of 56 mmol/mol (7.3 %). Only three of the twelve sequenced participants with diabetes had co-morbidities, most commonly retinopathy. Two participants also had nephropathy with stable kidney function. All type 1 diabetes participants used insulin, two used statins, while none used metformin or had immunosuppressive medications. Overall, the participants with type 1 diabetes were relatively well-controlled (Tables 1, S1, and S2). Whole blood cell counts were comparable between both groups (Tables S1 and S2).

**Table 1:** Participant characteristics

	HC (*n* = 12)^a^^,^^b^	DM (*n* = 12)^b^
Age, years	39.0 [25.0, 64.0]	40.0 [21.0, 62.0]
Female sex	4	4
BMI, kg/m^2^	23.9 [18.2, 29.1]	25.3 [20.0, 31.3]
HbA1c, mmol/mol	NA	56.0 [48.0, 108]
DM duration, years	NA	19.0 [7.00, 40.0]
Glucose, mmol/L	4.54 [3.47, 5.11]	NA
C-reactive protein, mg/L	1.44 [0.281, 8.16]	3.67 [0.454, 30.0]

Data are presented as number or median [min, max].

HC = healthy controls, DM = type 1 diabetes mellitus, BMI = body mass index, HbA1c = glycated hemoglobin, NA = not applicable.

a Additional participant characteristics can be found in Tables S1 and S2.

b Summarized characteristics based on participants included in the RNA-sequencing analysis. Sample number was different for the macrophage cytokine data analysis (*n* = 17).

#### Ethics statement

Blood was drawn from healthy volunteers after obtaining written informed consent, which was approved to be not subject to the WMO by the Medical Ethical Committee Oost-Nederland (NL 32357.091.10). Additional blood samples were collected from individuals with type 1 diabetes during routine blood draws, following the provision of written informed consent. This procedure was approved by the Medical Ethical Committee Oost-Nederland (2022-13621).

#### Macrophage culture

Peripheral blood mononuclear cells (PBMCs) were isolated from EDTA whole blood using Ficoll-Paque (GE Healthcare) density gradient centrifugation. After isolation, PBMCs were washed twice with phosphate-buffered saline (PBS) and resuspended in Dutch-modified RPMI 1640 medium (Invitrogen) supplemented with 50 μg/mL gentamicin, 2 mM Glutamax (GIBCO), and 1 mM pyruvate (GIBCO). PBMCs were seeded in non-tissue culture-treated Petri dishes (Corning) to isolate monocytes through plastic adhesion. Monocytes were cultured for six days in the presence of 5 ng/mL M-CSF (Miltenyi Biotec) and 10% human pooled serum to generate human monocyte-derived macrophages (hMDMs). Although use of monocyte-derived macrophages for ex-vivo *Mtb* infection is an established model, it does not translate directly to dynamics *in vivo*.

#### *Mycobacterium tuberculosis* strain

*Mycobacterium tuberculosis* H37Rv strain (ATCC) was cultivated to mid-logarithmic phase in Middlebrook 7H9 liquid medium (Difco, Becton-Dickinson) at 37°C. Growth medium was supplemented with 10% oleic acid–albumin–dextrose–catalase (BBL, Becton-Dickinson), 0.5% glycerol (Promega, Madison, WI, USA), and 0.05% Tween (Sigma, Saint Louis, MO, USA). Cultures were aliquoted in TSB containing 40% glycerol and stored at −80°C. For macrophage infections, frozen aliquots were thawed six days before the infection and were grown to mid-log phase in Middlebrook 7H9 liquid medium (Difco, Becton-Dickinson) supplemented with oleic acid/albumin/dextrose/catalase (OADC) (BBL, Becton-Dickinson).

### Method details

#### Infection

This study uses an early *in vitro* macrophage-*Mtb* infection model. HMDMs were infected with *Mtb* H37Rv strain at a multiplicity of infection of 5:1 for four hours. In short, macrophages were seeded in tissue culture-treated 6-well plates (Corning) at a density of 1x10^6^ macrophages per well for RNA-sequencing or in tissue-culture treated 96-well plates at a density 0,1x10^6^ macrophages per well for colony forming unit measurements. HMDMs were incubated overnight in antibiotic-free medium at 37°C in a 5% CO_2_ atmosphere. The next day, mid-log phase *Mtb* cultures were harvested by centrifugation, resuspended in PBS with 0,1% Tween (Sigma-Aldrich), passaged 10 times through a 21-gauge needle, centrifuged once more, and resuspended in PBS. *Mtb* was diluted to a 0.5 McFarland and then used to infect macrophages. After four hours of infection, supernatants were collected, filtered, and stored at -80°C. Extracellular bacteria were removed by washing adherent hMDMs with PBS.

#### RNA extraction

HMDMs were incubated for 10 minutes in RA1 lysis buffer (Macherey-Nagel) to release only the macrophage RNA into suspension. After scraping each well, the suspension was transferred into Eppendorf tubes containing glass beads. Tubes were centrifuged at 1500 x g for 10 minutes to pellet bacteria. 60% v/v of the supernatant was removed to partially dispose of the macrophage RNA (Figures 1A and 1B). Samples were bead-beaten twice for 30 seconds at 4300 rpm in a BeadBug (Benchmark Scientific) and once for 30 seconds at 7000 rpm in a MagNAlyser (Roche) to release *Mtb* RNA. Samples were transferred to RNA isolation columns without taking the glass beads along. Further RNA isolation was performed following the NucleoSpin RNA isolation Kit (Macherey-Nagel) instructions. RNA concentration was measured using the Qubit RNA high sensitivity assay and RNA integrity was analysed using the Agilent 2100 Bioanalyzer and the high sensitivity RNA screen tape and sample buffer (Agilent). Gel electrophoresis of extracted RNA clearly confirmed the presence of both prokaryotic 16S and 23S ribosomal RNA, as well as eukaryotic 18S and 28S ribosomal RNA, with distinct peaks observed for each (Figure 1C). RNA was stored at -80°C until the library preparation.

#### Library preparation, enrichment, and RNA-sequencing

Libraries were prepared following the Illumina Stranded Total RNA Prep, Ligation with Ribo-Zero Plus instructions (Illumina) including rRNA depletion. Concentration of the libraries was measured using Qubit dsDNA BR Assay Kit (Thermo Fisher) and library fragment size was checked using the Agilent 2100 Bioanalyzer and DNA 1000 screen tape and sample buffer (Agilent).

To enrich only for the *Mtb*-libraries, a myBaits Custom Hybridization Capture Kit (Daicel Arbor Biosciences) was used, including biotinylated RNA probes (baits), with sequences corresponding to the genome of *Mycobacterium tuberculosis* (Figure 1B). Baits included only coding sequences (CDS), excluding for example tRNAs, rRNAs, transposases, and other non-coding regions. Highly similar sequences to the human genome (> 95 %) were excluded to avoid hybridization to the exogenous human DNA in the samples. Library enrichment was performed following the MyBaits Hybridization Capture for Targeted NGS high-sensitivity protocol. Concentration and quality of final enriched *Mtb*-libraries was measured using Qubit dsDNA BR Assay Kit (Thermo Fisher) and library fragment size was checked using the Agilent 2100 Bioanalyzer and DNA 1000 screen tape and sample buffer (Agilent). The final library pool consisted of four times more hMDM library than *Mtb* library to correct for higher amplification rate of small *Mtb*-libraries. The library pool was sequenced paired-end on a SP cartridge on the NovaSeq 6000 with 2x100 cycles. Sequencing resulted in >2 million uniquely mapped read counts for bacterial samples and in >20 million read counts per human sample (Figures S1A and S1B).

#### Cytokine measurement

Cytokine levels present in the supernatant of uninfected and infected macrophages were assessed using a multiplex proximity extension assay (Olink Proteomics, Uppsala, Sweden). In this method, oligonucleotide-labeled antibodies bind to target proteins; when two antibodies bind in close proximity, they are linked and detected via DNA polymerase extension and real-time PCR. To reduce variability, samples were randomized across plates, and plasma from the uninfected and infected condition of the same participant and their matched control was analysed on the same plate. Absolute concentrations were used for the analysis. A total of 36 proteins were detectable in >75% of samples and included in the analysis. Samples were excluded if a QC warning coincided with strong batch effects in the principal component analysis (PCA).

#### Colony forming units

Colony-forming units (CFUs) were measured after four hours and 48 hours of infection. Extracellular CFUs were determined by plating the supernatant of hMDMs on Middlebrook 7H10 agar (Difco, Becton-Dickinson) supplemented with OADC. For intracellular CFUs, the supernatant was removed from wells, and hMDMs were lysed in water for 10 minutes before plating the lysate onto Middlebrook 7H10 agar with OADC. CFUs were counted after about 3 weeks of incubation.

#### Glucose assay

Circulating plasma glucose from healthy participants (*n* = 17) was measured using a Glucose assay kit (Abcam) to ensure a concentration within the healthy range.

#### C-reactive protein ELISA

Circulating c-reactive protein, a marker for systemic inflammation, was measured in plasma samples from healthy participants (*n* = 17) and individuals with type 1 diabetes (*n* = 17) using the Human C-reactive Protein/CRP DuoSet ELISA (Biotechne, R&D systems).

### Quantification and statistical analysis

#### Dual RNA-sequencing read alignment

RNA-seq reads from macrophages infected with *Mtb* were trimmed to remove adapter sequences and low-quality bases using fastp (version 0.23.4). Trimming parameters included removing bases with a Phred score <20 from the 3' end and discarding reads shorter than 25 base pairs. The trimmed reads were then aligned to the Homo sapiens reference genome (GRCh38) using STAR (version 2.7.3a) with default parameters, adjusting the --sjdbOverhang parameter to 101 according to the FASTQ read length in our study. A second alignment was performed against the *Mtb* H37Rv reference genome (NC_000962.3). The resulting BAM files for human alignment and *Mtb* alignment were used to quantify gene-level read counts using the GenomicAlignments package in R (version 4.1.2).

#### Differential gene expression analysis

Differential gene expression analysis was carried out in R using the DeSeq2 pipeline.^24^ We fitted the DeSeq2 negative binomial generalized linear model with FDR-corrected Wald test. We compared the paired infected versus uninfected macrophages from people with type 1 diabetes or healthy controls using this design formula: ∼ DonorID + Infection. When comparing macrophages from people with type 1 diabetes versus healthy controls within either the uninfected or infected state the following design formula was used: ∼ Experiment + Sex + Phenotype. Experiment and sex were included as they were separated in PC2 and PC3 (Figure S2B). Experiment and sex were not included in the paired analyses, because participants with type 1 diabetes and their healthy controls were sex- and age-matched and always processed simultaneously to prevent technical variability. To explicitly test whether there were any genes that showed a distinct response to infection in type 1 diabetes vs healthy controls we fit an interaction model in DESeq2. For this model, the following design formula was used: ∼ Phenotype + Phenotype:ID_special + Phenotype:Infection, with ID_special accounting for inter-individual differences within each group.

Genes with less than 12 counts in one comparison group were excluded from downstream analysis. Sample DM16_1 clustered with infected samples at both the transcriptional and protein level, despite being labelled as uninfected, suggesting contamination. As a result, participant DM16 was excluded from all paired analyses (Figures S2A and S2B). Pathway enrichment analysis for macrophage data was performed using the GO database.

Regarding the mycobacterial gene expression analysis, we compared intracellular *Mtb* inside macrophages from people with type 1 diabetes versus *Mtb* alone using ∼Condition. The same was done for intracellular *Mtb* inside healthy macrophages. Additionally, we compared intracellular *Mtb* in macrophages from people with type 1 diabetes versus intracellular *Mtb* inside macrophages from healthy controls. *Z*-scores between intracellular *Mtb* in macrophages from people with diabetes, intracellular *Mtb* in macrophages from healthy controls, and *Mtb* alone was calculated using the standard deviation of one group from the mean gene expression across all three groups. *Mtb* pathway enrichment analysis was done using CAMM categories,^25^ which were kindly provided by Elizabeth A. Wynn and Nicholas D. Walter from the University of Colorado Anschutz Medical Campus, Colorado, USA.

#### Transcriptional data integration of host and pathogen

To integrate host and pathogen transcriptional responses during infection, we performed FDR-corrected Spearman correlation of MSMD-associated host macrophage genes with the 500 most variable genes across all intracellular *Mtb* samples in macrophages from people with diabetes and controls. Rank-based spearman correlation with an adjusted *p*-value <0.05 was chosen to ensure robust analysis of non-normally distributed data and to keep the probability of type 1 error under 5%. The MSMD-associated genes were chosen, because mutations in them are known to cause susceptibility to mycobacterial diseases and are linked to IFN-γ, which appeared to be slightly different between macrophages from people with diabetes and controls. The most variable 500 intracellular *Mtb* genes were chosen, because the *Mtb* H37Rv strain cultured for all experiments is expected to be transcriptionally the same, and therefore, the most variable intracellular *Mtb* genes display any variability in the host environment best. Gene overrepresentation analysis was performed on *Mtb* genes with a spearman correlation >0.6 with the host gene *CYBB* in healthy macrophages using the GO database to identify biological function of these genes.

To further examine host-pathogen coordination in an unbiased, genome-wide manner, we applied weighted gene co-expression network analysis (WGCNA) independently to the infected host and intracellular *Mtb* transcriptomes, defining modules of co-expressed host and *Mtb* genes. For the WGCNA analysis, all macrophage genes with a gene count above 12 in six samples and all intracellular *Mtb* genes were included to calculate co-expression modules. WGCNA was done separately for people with type 1 diabetes and healthy controls. Eventually, macrophage module eigengenes were correlated with intracellular *Mtb* module eigengenes using FDR-adjusted Spearman's correlation to assess coordination between host and pathogen transcriptional programs at the network level. All analyses were performed using R version 4.5.0.

#### Cytokine data analysis

Absolute concentrations of measured cytokines were used for the analysis. Quality control was performed by excluding measurements with >25% missing values in both the uninfected and infected state and by testing for the presence of batch effects by using PCA. Three outliers (based on PCA and RNA-sequencing results) were removed: HC14-, DM15-, and DM16-, resulting in the following sample numbers: HC uninfected (*n* = 16), DM uninfected (*n* = 15), HC infected (*n* = 17), DM infected (*n* = 17). Measurements that passed quality control, were analysed by PCA stratified for phenotype and differences between groups were tested by using the FDR-corrected signed-rank two-sided pairwise Wilcoxon test (HC: infected vs. uninfected, DM: infected vs. uninfected) and an unpaired Wilcoxon test with FDR-correction when comparing phenotypes within one infection state (infected: DM vs. HC, uninfected: DM vs. HC). Results were illustrated in a combined plot showing log 2 fold changes of both phenotypes. All results were assigned to be significant if the adjusted *p*-value was <0.05. All analyses were performed using R version 4.5.0.
