# Supplementary material for: Type 1 diabetes alters early macrophage-Mycobacterium tuberculosis transcriptional coordination during infection
Source: iScience. 2026 Jun 23;29(7):116418. doi: 10.1016/j.isci.2026.116418 (PMC13320272; doi:10.1016/j.isci.2026.116418)
Supplement: Document S1. Figures S1–S6 and Tables S1 and S2 [file mmc1.pdf]

iScience, Volume *29*

Supplemental information

Type 1 diabetes alters early macrophage-*Mycobacterium tuberculosis* transcriptional coordination during infection

Julia Brake, Nicholas A. Sumpter, Valerie A.C.M. Koeken, Jodie A. Schildkraut, Eva Terschlüsen, Cees J. Tack, Edwin Ardiansyah, Vinod Kumar, Jakko van Ingen, and Reinout van Crevel

Supplementary Table 1: Participant characteristics from dual RNA-sequencing samples. *

	HC (n = 12)	T1DM (n = 12)
**Age, years**	39.0 [25.0, 64.0]	40.0 [21.0, 62.0]
**Female sex**	4	4
**BMI, kg/m^2**	23.9 [18.2, 29.1]	25.3 [20.0, 31.3]
**HbA1c, mmol/mol**	NA	56.0 [48.0, 108]
**DM duration, years**	NA	19.0 [7.00, 40.0]
**Glucose, mmol/L**	4.54 [3.47, 5.11]	NA
**Glycaemic burden****	NA	1170 [399, 2380]
**C-reactive protein, mg/L**	1.44 [0.281, 8.16]	3.67 [0.454, 30.0]
**Smoking**	0	2
**Neutrophils, %**	54.8 [42.1, 71.2]	55.2 [32.8, 73.2]
**Lymphocytes, %**	32.7 [14.0, 47.3]	33.2 [17.5, 59.0]
**Monocytes, %**	9.25 [6.50, 11.9]	7.05 [6.00, 8.70]
**Eosinophils, %**	1.90 [1.40, 6.10]	2.70 [0.900, 4.50]
**Basophils, %**	0.50 [0.20, 1.10]	0.70 [0.20, 1.20]

* Summarized participant characteristics based on participants, who are included in the RNA-sequencing analysis. Data are presented as number or median [min, max]. Abbreviations: HC = healthy controls, T1DM = type 1 diabetes mellitus, BMI = Body mass index, HbA1c = glycated haemoglobin, NA = not applicable.
**Glycaemic burden was calculated as HbA1c (%) * DM duration (years).

Supplementary Table 2: Participant characteristics from supernatant samples. *

	HC (n = 17)	T1DM (n = 17)
**Age, years**	34.0 [25.0, 64.0]	46.0 [21.0, 64.0]
**Female sex**	5	5
**BMI, kg/m^2**	23.5 [18.2, 29.1]	26.1 [20.0, 31.3]
**HbA1c, mmol/mol**	NA	59.0 [48.0, 108]
**DM duration, years**	NA	22.0 [7.00, 48.0]
**Glucose, mmol/L**	4.52 [3.47, 5.11]	NA
**Glycaemic burden****	NA	1390 [399, 3260]
**C-reactive protein, mg/L**	1.66 [0.280, 8.17]	3.62 [0.450, 30.0]
**Smoking**	1	4
**Neutrophils, %**	55.0 [42.1, 75.6]	55.9 [32.8, 73.2]
**Lymphocytes, %**	32.5 [12.9, 47.3]	32.8 [17.5, 59.0]
**Monocytes, %**	9.00 [6.50, 11.9]	7.00 [6.00, 8.70]
**Eosinophils, %**	1.80 [1.00, 6.10]	2.60 [0.40, 4.50]
**Basophils, %**	0.40 [0.20, 1.10]	0.70 [0.20, 1.20]

* Summarized participant characteristics based on participants from whom supernatant was available for Olink 48 cytokine panel analysis. Data are presented as number or median [min, max]. Abbreviations: HC = healthy controls, T1DM = type 1 diabetes mellitus, BMI = Body mass index, HbA1c = glycated haemoglobin, NA = not applicable.
**Glycaemic burden was calculated as HbA1c (%) * DM duration (years).

Fig. S1

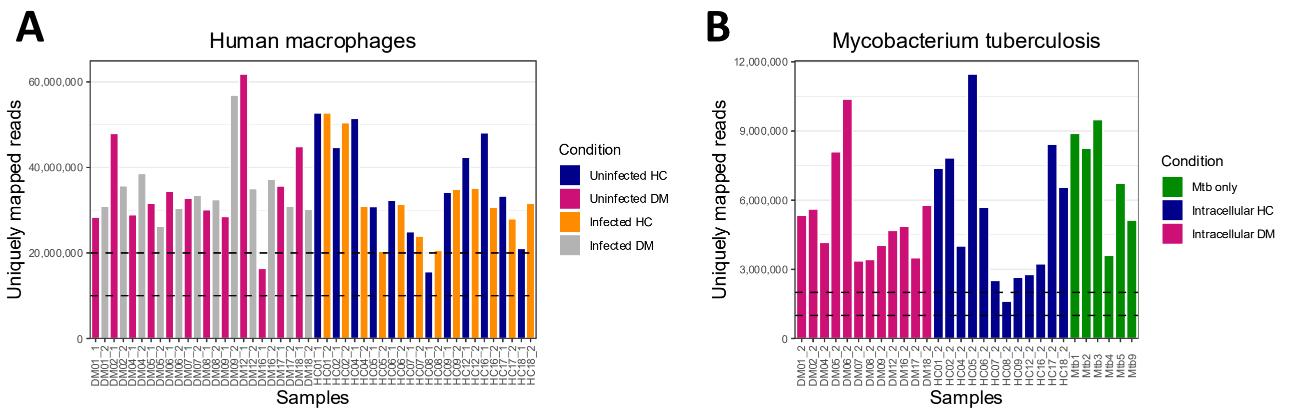

Figure S1. Read counts of all samples.

(A) Bar chart displaying the total number of reads per sample that uniquely mapped to the human hg38 genome for infected and uninfected macrophages from people with diabetes (grey, pink) and healthy controls (orange, blue). The lower dotted line marks ten million reads and the upper dotted line marks 20 million reads.

(B) Bar chart displaying the total number of reads per sample that uniquely mapped to the genome of *Mycobacterium tuberculosis* H37Rv for samples containing *Mtb* alone (green, n = 6), intracellular *Mtb* in macrophages from people with diabetes (pink) and controls (blue, n = 12 in both groups). The lower dotted line marks one million reads and the upper dotted line marks two million reads.

Fig. S2

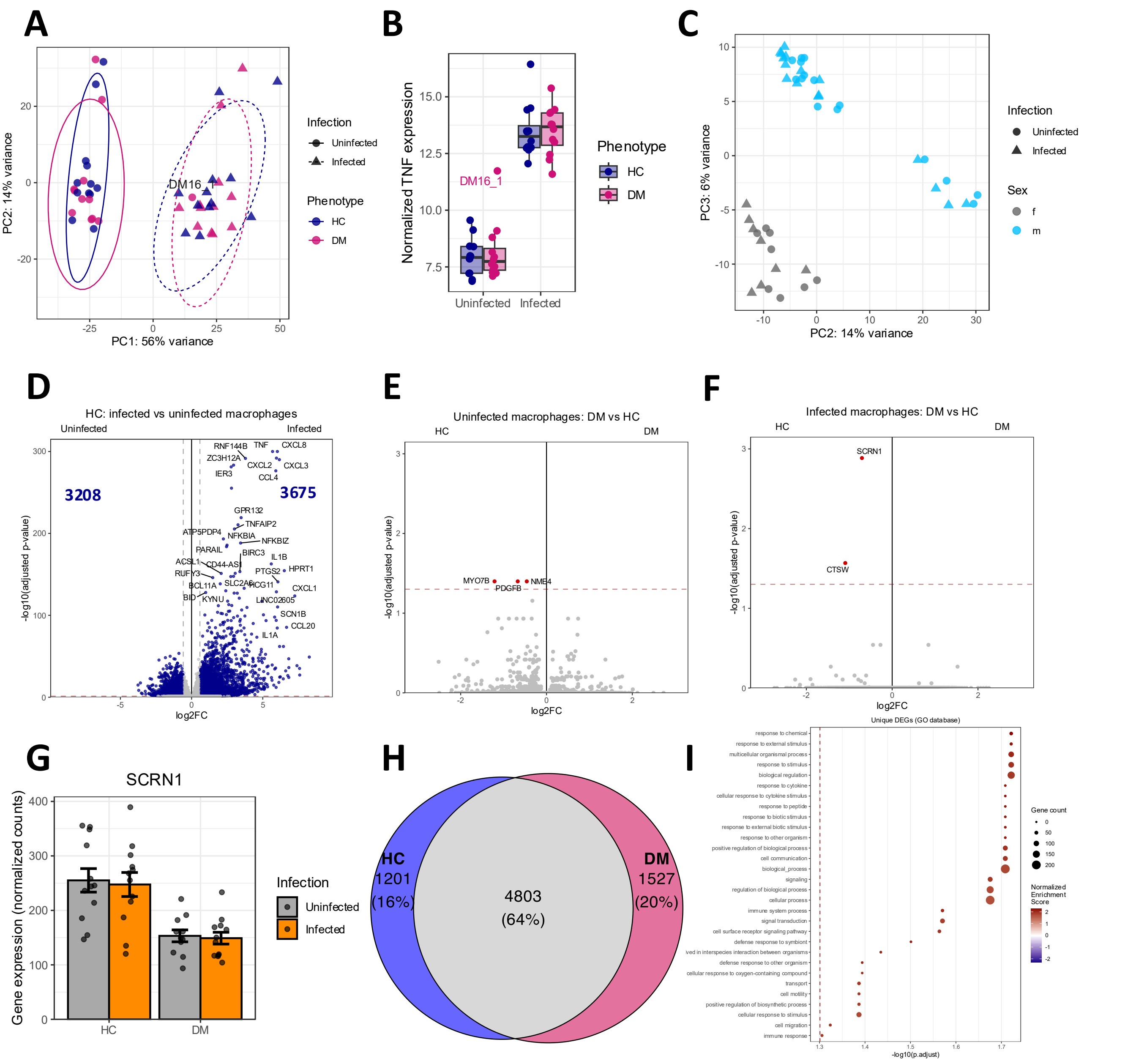

Figure S2. Transcriptional response of macrophages from people with diabetes and controls to *Mycobacterium tuberculosis*. **(A)** Principal-component analysis (PCA) plot showing the first two principal components of measured gene expression derived from uninfected (circles) and infected (triangles) macrophages from people with diabetes (pink) and control subjects (blue, n= 12 for both groups). Sample DM16_1 is labelled and was excluded from the analysis.

(B) Boxplot showing the normalized TNF gene expression for infected and uninfected macrophages from people with diabetes (n = 11) and healthy controls (n = 12). Sample DM16_1 is labeled and was excluded as an outlier.

(C) Principal-component analysis (PCA) plot showing the second and third principal components of measured gene expression derived from uninfected (circles) and infected (triangles) macrophages from people with diabetes (n = 11) and healthy controls (n = 12), showing female (grey) and male (turquoise) participants.

(D) Volcano plot comparing gene expression of infected and uninfected healthy control macrophages (n = 12), showing significance (-log10P) versus magnitude of change (Log2 fold change) of differentially expressed genes. Significant differences between infection state are depicted in blue (adjusted *p*-value <0.05, log2FC >0.585).

(E-F) Volcano plot comparing gene expression of macrophages from people with diabetes (n = 11) and controls (n = 12) either in the **(E)** uninfected or **(F)** infected state, showing significance (-log10P) versus magnitude of change (Log2 fold change) of differentially expressed genes. Significant differences between groups are depicted in red (adjusted *p*-value <0.05).

(G) Bar chart showing normalized gene counts for SCRN1 in uninfected (grey) and infected (orange) macrophages from people with diabetes (n = 11) and controls (n = 12).

(H) Venn diagram showing the DEGs upon *Mtb* infection (two-sided paired Wilcoxon Rank Sum test; adjusted *p*-value <0.05, log2FC >0.585), which are significant in both groups (grey), in macrophages from people with diabetes (grey), or in healthy control macrophages (blue) alone.

(I) Gene set enrichment analysis (GSEA) showing the most enriched pathways upon infection for genes that were only significant DEGs in healthy macrophages, ranked by significance (-log10(adjusted *p*-value)). The red dotted line represents the threshold of significance (adjusted *p*-value <0.05). Size of the bubbles reflects the gene ratio of the pathway (hit gene count/total gene count), and the color reflects the normalized enrichment score (red = positive, blue = negative).

Fig. S3

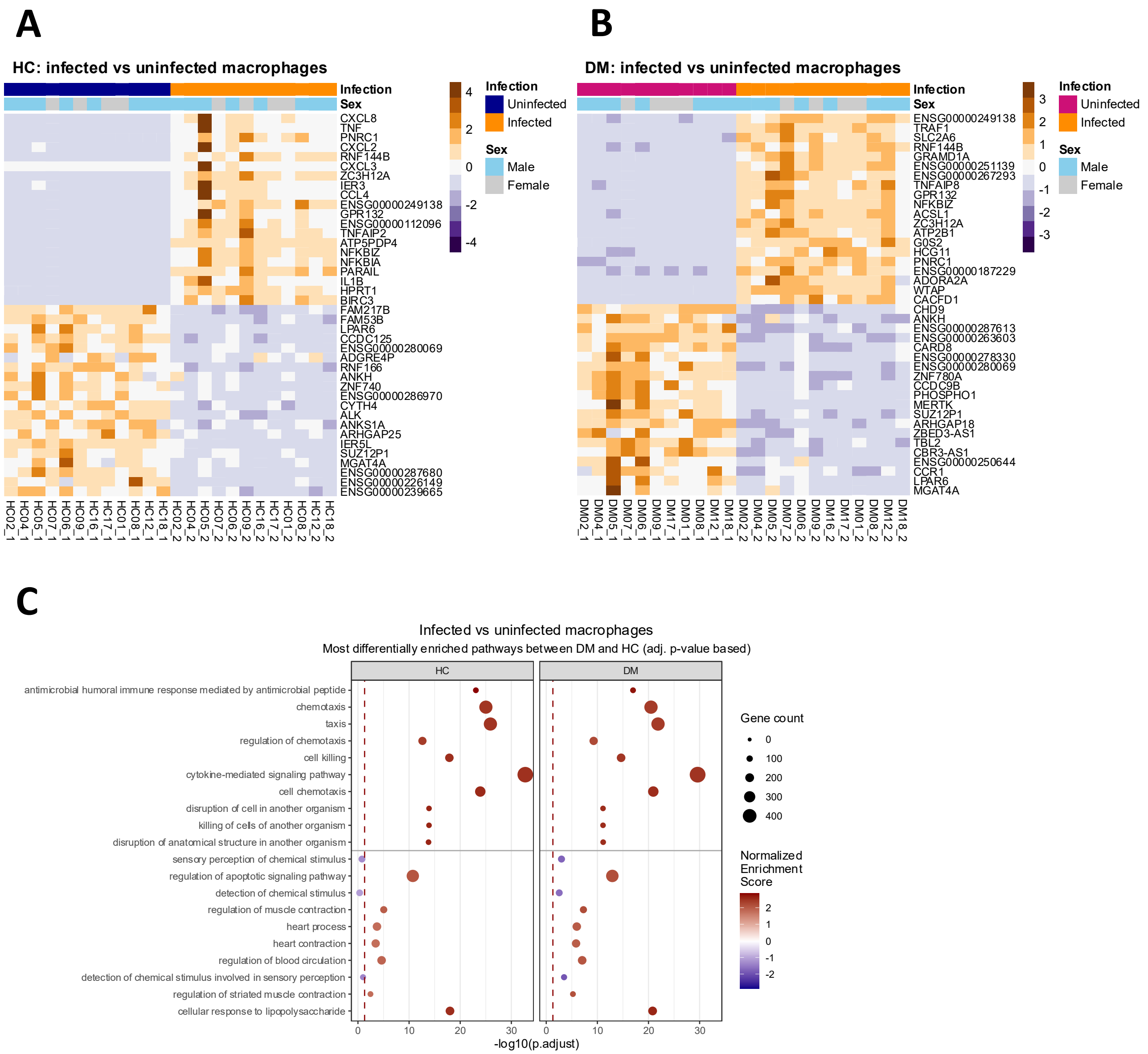

Figure S3. Top differentially expressed genes and pathways of macrophages from people with diabetes and controls upon *Mycobacterium tuberculosis* infection.

(A-B) Heatmap showing the top 20 up- and downregulated genes upon *Mtb* infection for **(A)** healthy control macrophages (n = 12) and **(B)** macrophages from people with diabetes (n = 11). Mean-based *z*-scores per gene across samples is shown (*z*-scores >0 = orange, <0 = blue).

(C) Gene set enrichment analysis (GSEA) showing the 20 most differentially enriched pathways upon infection between macrophages from people with diabetes and controls based on the adjusted adjusted *p*-value. Size of the bubbles reflects the gene ratio of the pathway (hit gene count/total gene count) and the color reflects the normalized enrichment score (red = positive, blue = negative).

Fig. S4

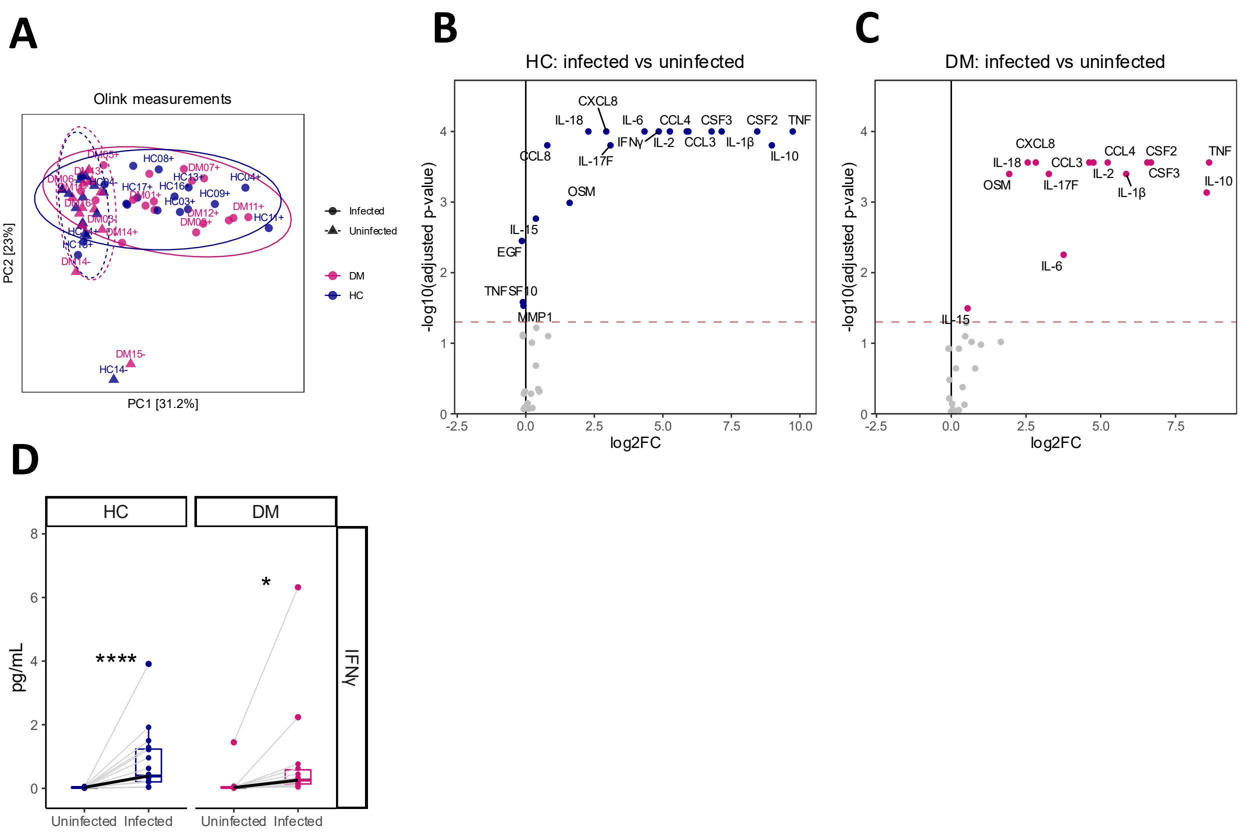

Figure S4. Cytokine levels of macrophages from people with diabetes and controls upon *Mycobacterium tuberculosis* infection.

(A) Principal-component analysis (PCA) plot showing the first two principal components of measured cytokines from infected (circles) and uninfected (triangles) macrophages from people with diabetes (pink) and controls (blue, in both groups n = 17). Outliers DM15- and HC14- are labeled and were excluded. Additionally, DM16- was excluded based on the RNA-seq results Final sample sizes: diabetes (uninfected: n = 15, infected: n = 17) and healthy controls (uninfected: n = 16, infected: n = 17).

(B-C) Volcano plot comparing cytokine concentrations of infected and uninfected **(B)** healthy control macrophages (blue, n = 16) and **(C)** macrophages from people with diabetes (pink, n = 15), showing significance (-log10P) versus magnitude of change (Log2 fold change) of differentially expressed genes. Significant differences between groups are depicted in blue or pink, respectively (two-sided paired Wilcoxon Rank Sum test; adjusted *p*-value <0.05).

(D) Boxplot showing the absolute concentrations (pg/mL) of IFN-γ in supernatants of infected and uninfected macrophages from people with diabetes (pink, n = 14) and controls (blue, n = 16) after exclusion of one paired diabetes sample that showed a decrease of IFN-γ upon infection (two-sided paired Wilcoxon Rank Sum test; adjusted *p*-value <0.05). ∗ adj. $p < 0.05$, ∗∗ adj. $p < 0.01$, ∗∗∗ adj. $p < 0.001$.

Fig. S5

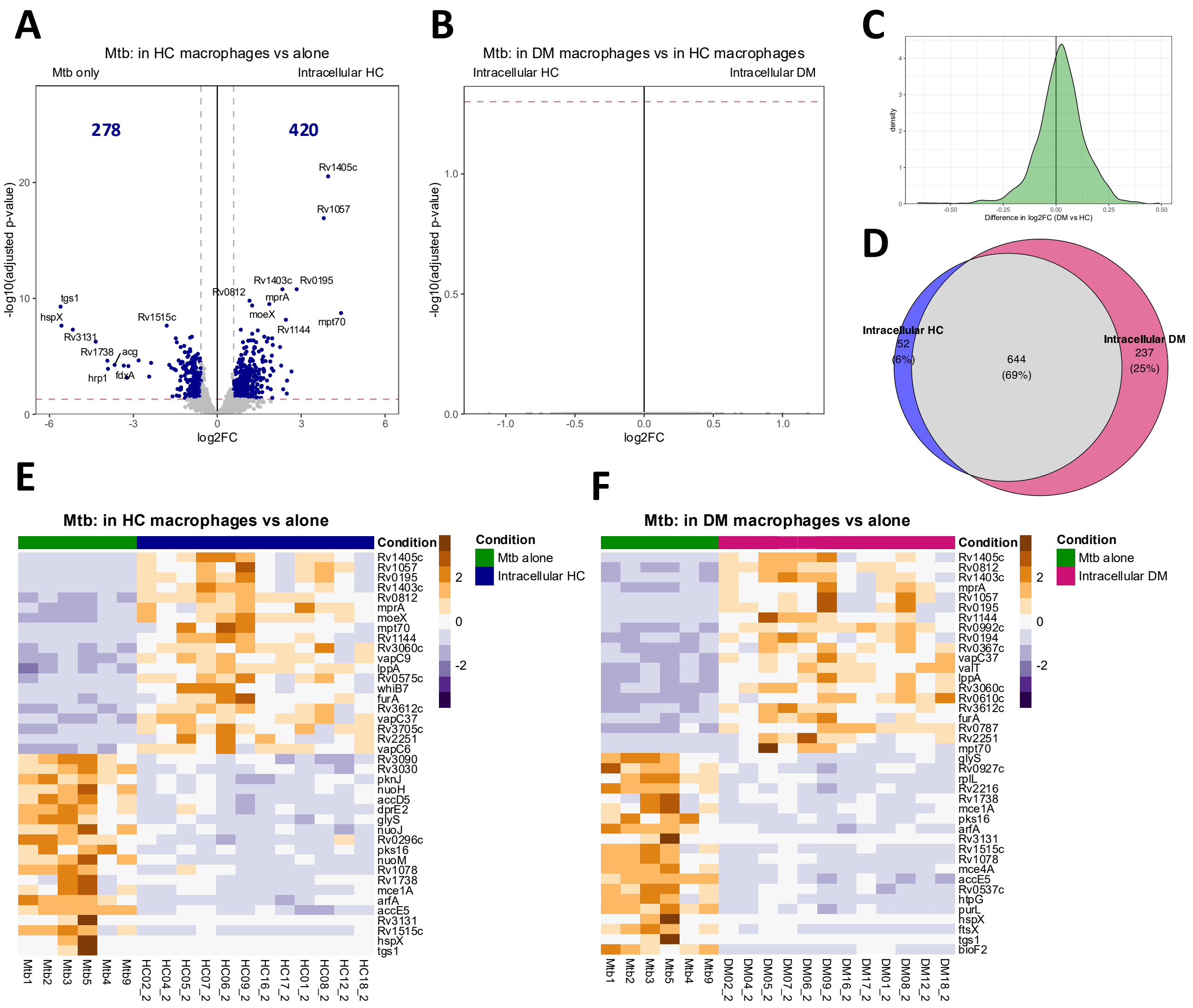

Figure S5. Transcriptional response of intracellular *Mycobacterium tuberculosis* in macrophages from people with diabetes and controls.
(A) Volcano plot comparing gene expression of *Mtb* alone (n = 6) versus being intracellular in healthy macrophages (n = 12), showing significance (-log10P) versus magnitude of change (Log2 fold change) of differentially expressed genes. Significant differences are depicted in blue (adjusted *p*-value <0.05).
(B) Volcano plot comparing gene expression of intracellular *Mtb* in macrophages from people with diabetes (n = 12) versus in control macrophages (n = 12), showing significance (-log10P) versus magnitude of change (Log2 fold change) of differentially expressed genes. Significant differences are depicted in red (adjusted *p*-value <0.05).
(C) Density plot of the difference in log2FC for all intracellular *Mtb* genes between macrophages from people with diabetes and controls.
(D) Venn diagram showing the intracellular *Mtb* DEGs (adjusted *p*-value <0.05, log2FC >0.585), which are significant in both macrophage groups (grey), in macrophages from people with diabetes (pink), or controls (blue).
(E-F) Heatmap showing the top 20 up- and downregulated genes for **(A)** *Mtb* in healthy control (n = 12) and **(B)** *Mtb* in macrophages from people with diabetes (n = 12). Mean-based *z*-scores per gene across samples is shown (*z*-scores >0 = orange, <0 = blue).

Fig. S6

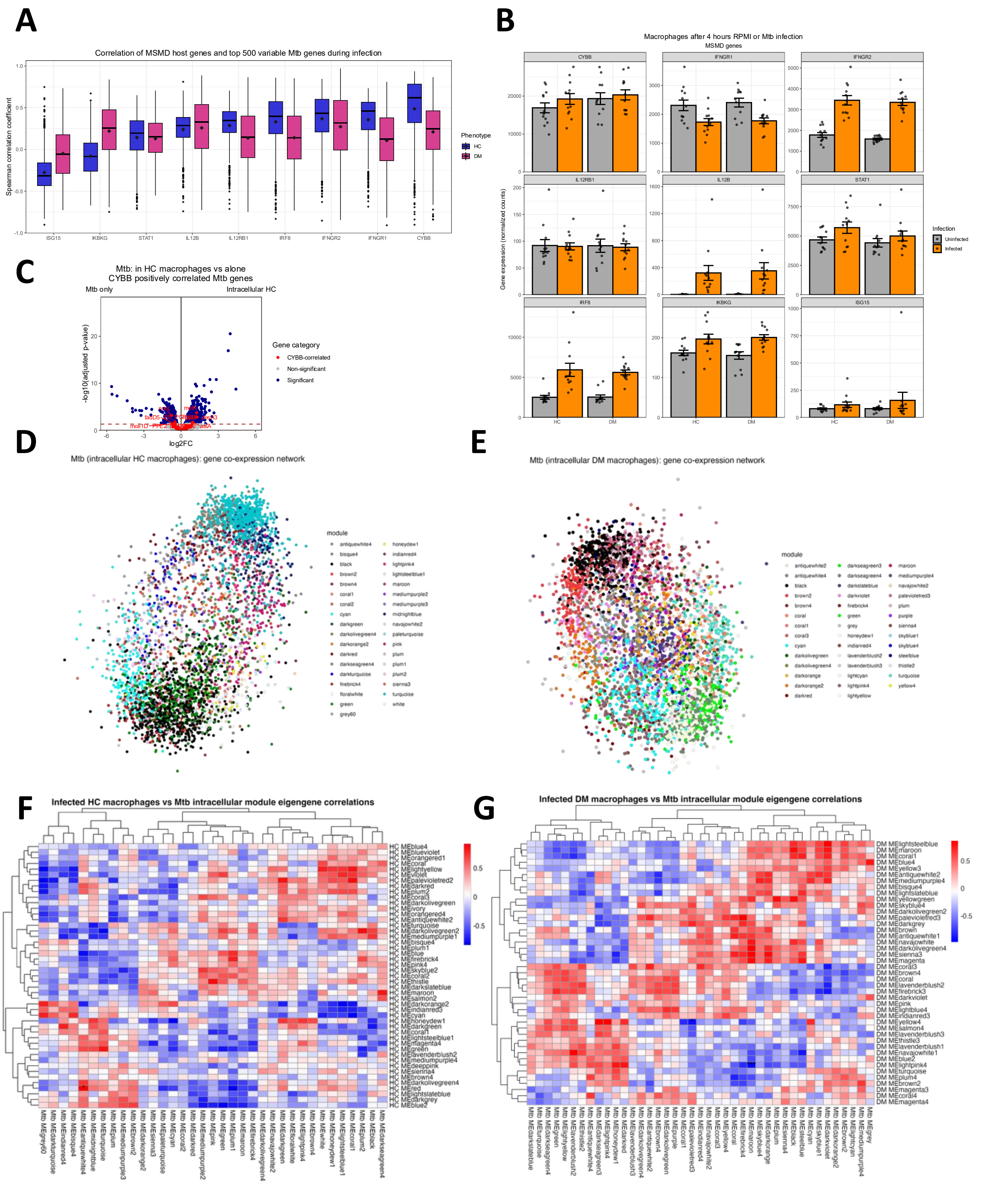

Figure S6. Host-pathogen transcriptional integration with weighted gene co-expression network analysis.
(A) Boxplot showing the average (diamond) and median (line) Spearmen correlation coefficient of host MSMD-genes with the 500 most variable intracellular *Mtb* genes for macrophages from people with diabetes (pink, n = 12) and controls (blue, n = 12).
(B) Bar charts showing the normalized read counts for MSMD-genes of infected (orange) and uninfected (grey) macrophages from people with diabetes and controls.
(C) Volcano plot comparing gene expression of healthy control macrophages (n = 12) before and after four hours of *Mtb* infection, showing significance (-log10P) versus magnitude of change (Log2 fold change) of the differentially expressed genes. Significant differences between infection state are depicted in blue (adjusted *p*-value <0.05). The with healthy control *CYBB* positively correlating intracellular *Mtb* genes, which were shown in the Spearman correlations in Figure 5A-B are colored in red.
(D-E) Weighted gene co-expression networks of **(D)** intracellular *Mtb* in healthy macrophages (n = 12) and **(E)** intracellular *Mtb* in diabetic macrophages (n = 11), showing the genes of each cluster separated by color.
(F-G) Spearman correlation of the module eigengenes of **(F)** healthy macrophages and **(G)** macrophages from people with diabetes with the corresponding intracellular *Mtb* module eigengenes (correlation coefficient >0 = red, <0 = blue; adjusted *p*-value <0.05). $*$ adj. $p < 0.05$, $**$ adj. $p < 0.01$, $***$ adj. $p < 0.001$.
